# Are digital interventions for smoking cessation in pregnancy effective? A systematic review protocol

**DOI:** 10.1186/s13643-016-0390-6

**Published:** 2016-12-01

**Authors:** Sarah Ellen Griffiths, Katherine E. Brown, Emily Anne Fulton, Ildiko Tombor, Felix Naughton

**Affiliations:** 1 Centre for Technology Enabled Health Research, Faculty of Health and Life Sciences, Coventry University, Richard Crossman Building, Priory Street, Coventry, CV1 5FB UK; 2 Health Behaviour Research Centre, Department of Epidemiology and Public Health, University College London, 1-19 Torrington Place, London, WC1E 6BT UK; 3 School of Health Sciences, University of East Anglia, Edith Cavell Building, Norwich, NR4 7UL UK

**Keywords:** Systematic review, Protocol, Smoking, Pregnancy, Digital interventions, M-health, e-health

## Abstract

**Background:**

Behavioural support for smoking cessation in pregnancy can be effective; however, many pregnant women face barriers to seeking support to stop smoking. Some digital interventions have been found to be effective for smoking cessation in the general population and may be effective for supporting cessation in pregnancy due to their flexibility and the potential for personalisation. To date, there is limited evidence of the effectiveness of digital interventions for smoking cessation in pregnancy. This review aims to assess the following: (1) whether digital interventions are effective at promoting smoking cessation among pregnant women; (2) which behaviour change techniques (BCTs) or combinations of BCTs are associated with the effectiveness of digital interventions for smoking cessation in pregnancy; and (3) whether the number of BCTs used is associated with the effectiveness of digital interventions for smoking cessation in pregnancy.

**Methods:**

This review will include digital interventions delivered largely through computer (PC or laptop), video/DVD, mobile phone (including smartphones) or portable handheld device (e.g. tablet, iPad) and include websites, mobile or tablet applications and SMS text messages. Interventions must be randomised or quasi-randomised controlled trials aimed at women who smoke in pregnancy, with smoking cessation as a measured outcome (preferably the latest available point prevalence smoking status measure taken during pregnancy, biochemically verified if available). Electronic bibliographic databases will be searched to identify suitable studies indexed in the following: Academic Search Complete, ASSIA, CINAHL, The Cochrane Library, EMBASE, Medline, PsycINFO, Scopus, and Web of Science. The search strategy will include key words and database-specific subject headings relating to ‘pregnancy’ and ‘smoking’ and synonyms for the terms ‘digital’ and ‘randomised controlled trial’. Where required and where possible, the first and second authors will independently code interventions and control groups for BCTs. If data allows, meta-analyses will be used to assess intervention effectiveness and the effectiveness of BCTs.

**Discussion:**

This systematic review will provide a detailed synthesis of the effectiveness of current research using digital interventions for smoking cessation in pregnancy, to build on the evidence base and guide the development of future research in this area.

**Systematic review registration:**

PROSPERO CRD42016036201

**Electronic supplementary material:**

The online version of this article (doi:10.1186/s13643-016-0390-6) contains supplementary material, which is available to authorized users.

## Background

Smoking in pregnancy is associated with a range of adverse pregnancy outcomes, including placental complications, spontaneous abortion, foetal growth restriction and low birth weight [1]. Prenatal exposure to tobacco smoke increases the risks of still-birth [2] and congenital birth defects, such as cardiovascular, musculoskeletal and limb reduction defects [3]. Prenatal smoking also increases the risks of childhood respiratory problems, including recurrent wheeze and asthma [4], and of developing nicotine dependence in adulthood [5]. Despite a general reduction in pregnancy smoking rates in high-income countries since the 1980s, this decline is not falling at the same rate across all social groups [6]. Women from socially disadvantaged groups are more likely to experience barriers to stopping smoking in pregnancy, such as perceiving smoking to be the only way of coping with stress and being influenced by their peers [7], and are thus less likely to quit successfully before giving birth [8]. Smoking during pregnancy remains a global health issue with huge variation in prevalence across and within countries. In the USA, the rate of smoking during pregnancy ranges across states from 1.8% in California to 27.1% in West Virginia [9]. In England, the rates of smoking at time of delivery also vary from 25.8% in South Tyneside, north-east England, to 1.4% in West London [10]. Across northern Europe, the rates of smoking in early pregnancy have been reported to vary from 12.5% in Denmark, 16.5% in Norway, 15% in Finland and 6.9% in Sweden [11].

Psychosocial interventions for smoking cessation in pregnancy can help to increase quit rates in the latter stages of pregnancy and can reduce the risk of low birth weight and preterm birth by up to 18% [12]. Other interventions, such as self-help [13], financial incentives [14], telephone support [15], and pharmacological interventions [16] have also demonstrated some efficacy for reducing smoking in pregnancy. In the UK, pregnant women are provided with free behavioural support to stop smoking through the National Health Service (NHS). Pregnancy specific support is provided by up to 67% of NHS services in England, with an average 4-week quit rate of 45.5% [17]. However, attendance at these services is low, and a steady drop in quit rates has been seen since 2011/2012 [10, 18]. Fear of being judged, an issue of time constraints and worry about failure have been reported as common barriers to attending such services [19, 20]. Lack of knowledge regarding the benefits, general ethos and accessibility of services have also been identified as barriers to accessing Stop Smoking Services [21]. This illustrates a need to consider alternative approaches to providing smoking cessation support for pregnant women [20].

There are currently 3.2 billion internet users and more than 7 billion mobile phone subscriptions worldwide [22]. Due to substantial improvements in public access to usable technology, digital interventions, or interventions which are mainly based around telephone, video, internet or mobile application technologies [23], have recently grown in popularity for promoting behaviour change. Whittaker et al. [24] report that mobile phone-based interventions for smoking cessation, primarily delivering support by text messages, increase the odds of abstinence at 6 months by 70% compared to those who did not receive the intervention. Similarly, internet interventions can be effective as an aid for smoking cessation, particularly if they are interactive and tailored to meet individual needs [25, 26]. Smartphone applications for smoking cessation are becoming increasingly popular [27]. However, the majority of existing smartphone apps and Facebook apps for smoking cessation rarely adhere to evidence-based practice [27–29]. A content analysis of the use of self-determination theory (SDT) in smartphone apps for smoking cessation reported that many current apps may also be missing the theoretical underpinning likely to make them most effective in the long term [30]. Recent randomised controlled trials have used small sample sizes and produced inconclusive results when compared to other digital interventions, such as text message interventions [31], making it difficult to assess the full effectiveness of smartphone apps for general smoking cessation [31, 32].

Digital interventions for smoking cessation in the general population can be beneficial. However, smoking in pregnancy may not be fully comparable to smoking in the general population. Due to physiological changes during pregnancy, such as blood levels of nicotine decreasing faster leading to more frequent nicotine withdrawal, quitting whilst pregnant may be even more difficult [33]. Smoking in pregnancy is closely linked to determinants of low socioeconomic status such as education, income, employment and social support networks [34]. Prenatal smoking is often used as a stress management resource, providing brief moments of relaxation in stressful lives and situations [7], particularly as pregnant women are likely to face internal and external pressure to stop smoking for the health of their baby [35]. In light of these issues, it is necessary to treat smoking in pregnancy as a separate issue from smoking in the general population and to collate research which specifically targets smoking in pregnancy.

The technological features of digital interventions make them relatively easy to tailor to the needs of the individual [36], which is likely to contribute to the effectiveness of interventions to aid smoking cessation in pregnancy [19, 20, 37, 38]. Digital interventions may also have the potential to address barriers that pregnant women face regarding seeking support to stop smoking, such as fear of failure and fear of being judged [19], and the accessibility of services [19, 20, 39]. As a result, digital interventions, such as SMS text message programmes (e.g. ‘MiQuit’, [40]) and mobile phone applications (e.g. ‘SmokeFree Baby’, [41]) have been developed for smoking in pregnancy. A recent review on the content and effectiveness of text message and app-based interventions for smoking cessation in pregnancy summarised programme characteristics and functions of available reviewed literature and programmes in-progress [42]. However, this review shows limited effectiveness data, reporting abstinence outcomes from only two text message programmes. Whilst demonstrating the promise of these interventions for smoking cessation in pregnancy, there remains a lack of evidence evaluating the effectiveness of such digital interventions. This highlights the need to collate research across all types of digital interventions for smoking cessation in pregnancy to ascertain whether these types of interventions are effective and to identify which approaches show most promise.

As highlighted in a review by Lorencatto et al. [43], there is limited reporting of the specific components, or behaviour change techniques (BCTs), that make up interventions in current literature reviews. BCTs are the smallest replicable components of an intervention, which can be used individually or in combination to alter or redirect the processes which are fundamental to behaviour change [44]. Specifying and reporting BCTs are important for enabling the accurate replication of effective interventions [44]. Lorencatto et al. [43] specified the BCT content of effective behavioural interventions for smoking cessation in pregnancy, using the smoking cessation taxonomy [45], which includes ‘provide information on the consequences of behaviour’ and ‘identifying barriers and/or problem solving’. A review on internet-based interventions for health promotion in the general population reported that theory-based interventions and interventions integrating more BCTs achieved larger effects [46]. However, a review of interventions addressing smoking, healthy eating and physical activity that specifically targeted low-income groups found that effective interventions tended to use fewer BCTs than ineffective interventions [47]. This contrast in evidence highlights how examining which BCTs have been used in digital interventions for pregnant smokers, and exploring whether there is a relationship between the number of BCTs used and intervention effectiveness will enhance the current research.

This review aims to address the current gap in the literature by analysing the use of digital interventions for smoking cessation in pregnancy. This will be done by synthesising the range of digital interventions currently being implemented and evaluating their effectiveness. To address the lack of research examining the mechanisms of these interventions, the BCT content of included papers will be explored where possible. For this review, we will be using the most up-to-date taxonomy: BCT Taxonomy v1, developed by Michie et al. [44].

### Objectives

This review aims to answer the following three research questions relating to digital interventions for smoking cessation in pregnancy.

Primary focus:

1. Are digital interventions more effective in increasing smoking cessation rates in pregnancy than usual care/other control groups?

Secondary focus:

2. Which BCTs/combinations of BCTs are associated with the effectiveness of digital interventions for smoking cessation in pregnancy?
3. Is the number of BCTs used associated with the effectiveness of digital interventions for smoking cessation in pregnancy?

## Method

The PRISMA-P guidelines for systematic review protocols have been followed for this protocol [48] (see Additional file 1).

### Eligibility criteria

Randomised and quasi-randomised controlled trials (including randomised pilot studies) will be included. Any type of comparison group will be included. Usual care for smoking cessation in pregnancy is typically brief smoking cessation advice provided by a health professional; however, any method of usual care will be acceptable for this review. If possible, trials using the same method of usual care will be pooled into a subgroup meta-analysis. If meta-analysis is not appropriate, usual care will only be analysed in a qualitative synthesis. Trials with more than one comparator will be included if at least one of the experimental arms meets the digital intervention inclusion criteria, as specified below.

For the purposes of this review, digital interventions will include any intervention delivered largely through a computer (PC or laptop), video or DVD, mobile telephone or portable handheld device (e.g. tablet, iPad). This includes, although is not limited to, email, videos, DVDs, websites or web-based games, mobile or tablet applications and SMS text messages or MMS multimedia messages. Interventions must be aimed at women who smoke in pregnancy, with smoking cessation as a measured outcome. These can be either delivered directly by an external source, e.g. health professional, researcher, peer, or family, or they can be self-administered. Interventions which include non-digital elements will be included only if the digital component is the primary element of the intervention and if any interaction with a health professional/counsellor/researcher etc. is purely to explain how the digital intervention works. Interventions which have been converted directly from a non-digital format (e.g. self-help leaflets in digital form) will be excluded.

Participants must be pregnant women at any stage of pregnancy, who have reported currently smoking cigarettes. As digital interventions designed to aid smoking cessation in younger pregnant adolescents are likely to be tailored to the specific needs of this age group, interventions focused specifically on participants under the age of 16 will be excluded. However, interventions designed for adults with no lower limit on age for enrolment will be included. Any studies with only women who have already quit smoking will be excluded. There will be no timing restrictions on publications. Articles must be written in English.

### Outcome measures

The preferred primary outcome will be the latest available point prevalence smoking status measure at the end of pregnancy, biochemically validated where possible or self-reported, as this is the most common measure used in smoking cessation literature. Prolonged abstinence from a set quit date will also be acceptable, preferably biochemically validated if available but self-report if not, so as not to exclude relevant studies reporting the effectiveness of a digital intervention on smoking abstinence in pregnancy.

Secondary data outcomes, if reported, include process measures relating to smoking cessation, such as setting a quit date and self-efficacy, and outcomes relating directly to the intervention, such as intervention use.

Secondary, descriptive outcomes will be BCT content of interventions and control groups. Where this information is not provided in the text or appendices of papers, the research authors will be contacted to ask whether this information is available. If not, authors will be asked for permission to code the relevant manuals for the BCTs used, or coding of the intervention description in the manuscript will be carried out.

### Information sources

Electronic bibliographic databases will be searched between August 2016 and October 2016: Academic Search Complete, ASSIA, CINAHL, The Cochrane Library, EMBASE, Medline, PsycINFO, Scopus and Web of Science. Both key words and database-specific subject headings relating to ‘pregnancy’ and ‘smoking’, variations of the term ‘digital’ covering computer, video, internet, app, telephone and mobile phone, and variations for ‘randomised control trial’ will be searched. Boolean logic using AND OR will be employed where appropriate.

Reference lists of studies identified for the review and relevant published reviews will be searched by hand. Authors of included studies will be contacted to determine whether they are aware of any relevant, unpublished articles that meet the inclusion criteria. Papers citing included studies will also be examined. Authors may also have to be asked for access to the intervention if access is not provided in the paper. This would be to enable two review authors to independently code the interventions and usual care/control group for BCTs, if possible, using the BCT Taxonomy v1 developed by Michie et al. [44], if this has not already been done. If this is not possible, BCT content will be inferred by coding the intervention description given in the manuscript.

The following research registers will be searched using the inclusion criteria for recently completed, unpublished clinical trials: National Institute for Health Research UK Clinical Trials Gateway, ClinicalTrials.gov, and Current Controlled Trials through the ISRCTN registry.

### Search strategy

An information specialist has provided support for this work to ensure the most exhaustive search terms have been employed. An example of the CINAHL database search strategy is provided in Additional file 2. This will be amended for other databases using database-specific subject headings, and keywords in both titles and abstracts will be searched.

### Data management and screening process

Data will be managed using EndNote software. Results from the original search will be combined and alphabetised, and duplications will be removed. The first author (SG) will then independently screen all titles and/or abstracts for the first phase of the review. A second reviewer (KB) will perform a calibration exercise by screening the first 100 titles and/or abstracts, or 10% of results (whichever is greatest), using a checklist to identify relevant papers. The Kappa statistic will be calculated to measure agreement [49]. If this is below 0.80, which can be used as a mark of strong agreement [50], a second exercise will be carried out on a further 50, or 5% of results, and any remaining discrepancies will be resolved following group discussion. For the second phase, full-text reports of potentially relevant studies will be obtained and independently checked against the inclusion criteria checklist by SG and another reviewer. Any disagreement over the suitability of a paper will be discussed with a third reviewer until consensus is reached. Data extraction and assessment of eligibility of studies for meta-analysis will then be carried out independently by SG and another reviewer using a data extraction sheet for the third phase. Any discrepancies will be discussed with a third reviewer if agreement is not reached.

### Quality assessment

The Cochrane Collaboration’s tool for assessing risk of bias in randomised trials will be used by SG and another reviewer and will be adapted to assess the validity of the studies included in the review [51]. Studies will be assessed against the criteria below and given either a classification of low risk, medium risk or high risk of bias:

1. Sequence generation
2. Allocation concealment
3. Blinding of participants, personnel and outcome assessors
4. Incomplete outcome data
5. Selective outcome reporting
6. Other sources of bias

### Data extraction and synthesis

Key details from each paper which meets the inclusion criteria will be extracted and synthesised into two groups:

1. Descriptive details of interventions that have been developed for smoking cessation in pregnancy, including study details, participant information, mode of delivery and intervention and control group details.
2. Details of the intervention content, which will be specified by respective BCTs (where possible). Coding will follow guidelines from Michie et al. [44] by SG and another reviewer independently. For studies using a control group, this will also be coded for BCTs to establish whether BCTs used in the intervention are specific to the intervention itself. Any disagreements will be discussed until a consensus is reached. Extracted data will then be synthesised. If the research team are unable to contact the study author and the manuscript does not provide enough detail for coding BCTs, the study will only be included in the initial meta-analysis of pooled trials, if suitable.

### Data analyses

#### Measures of treatment effect

The number of participants reporting to be abstinent from each trial will be extracted and presented as odds ratios as they have advantageous mathematical properties compared to risk ratios when dealing with low event rates and are more commonly reported in smoking cessation literature. An intention to treat basis will be applied, where any missing participant data will be assumed to be smokers (non-abstinent).

#### Statistical analysis

To address the primary objective relating to the effectiveness of digital interventions, if appropriate, a meta-analysis will be carried out pooling effect sizes of trials comparing a digital intervention to usual care or control group. In order to carry out a meta-analysis, there will need to be similar RCTs meeting the inclusion criteria: studies must be focused on pregnant women, must be an RCT or quasi-randomised controlled trial, must be a digital intervention, must have a control group and smoking cessation must be reported as an outcome. Studies must also report event rates for effect size calculation. If the data from the intervention is not appropriate for meta-analysis, only a narrative synthesis will be carried out.

If data are suitable for meta-analysis, subgroup meta-analyses will be carried out pooling studies which have used particular BCTs, where it has been possible to code for BCT content. This will address the second objective regarding which BCTs or groups of BCTs are associated with the effectiveness of digital interventions for smoking cessation in pregnancy.

To address the third objective, if meta-analysis is deemed possible, a logistic meta-regression will be used to explore if a relationship exists between the number of BCTs used and intervention effectiveness. Regression coefficients and their test of significance will be reported. If BCT data is not suitable for meta-analysis, this information will only be included in the narrative synthesis.

#### Heterogeneity

As interventions will differ, effects are considered to fall on a distribution of effect sizes; therefore, a random effects model will be adopted for the meta-analyses, estimating intervention effects with 95% confidence intervals (CIs) and significance at the 5% level. Heterogeneity between studies will be assessed using the chi-squared test and, if significant, analysis of the inconsistency index (*I* ^2^) will measure how much variation across studies is a result of heterogeneity, rather than chance [52]: where *I* ^2^ is more than 50%, this will indicate significant heterogeneity.

#### Publication bias

Funnel plots and tests of asymmetry will be used to assess publication bias [53]. If publication bias is detected, Duval and Tweedie trim and fill statistics will be used to adjust the effect size for missing studies.

#### Sensitivity analysis

Anticipated sensitivity analyses include limiting the primary analysis to biochemically validated outcomes only if possible and excluding any trials considered borderline with respect to the inclusion criteria.

#### Summary of findings table

GRADE system principles will be used to assess the quality of evidence regarding reported outcomes [54], and a summary of findings table will be produced. Outcomes are expected to include the latest available point prevalence abstinence during pregnancy, process measures such as setting a quit date and self-efficacy, other outcomes relating directly to the intervention, such as intervention use, and BCT content of interventions and control groups, if data is available.

## Discussion

The use of digital technology for smoking cessation in pregnancy is a new and expanding field, which will benefit from the detailed synthesis of research proposed in this review. Exploring the effectiveness of digital interventions, and the use of and effectiveness of behaviour change techniques, will provide a necessary addition to the current research-base for smoking cessation in pregnancy

### Limitations

The novelty of this field may result in a restricted number of studies meeting the inclusion criteria; included studies may be small-scale or pilot RCTs using limited sample sizes. However, it is expected that there will be enough research to allow for a detailed exploration of the effectiveness of a variety of digital interventions for smoking cessation in pregnancy.

As issues have been raised regarding the accessibility of some forms of digital interventions, such as the use of websites and smartphone apps, among pregnant smokers from lower socioeconomic backgrounds [55], it would be beneficial to explore this in a quantitative analysis. Unfortunately, it is unlikely that the data from this review will allow for a quantitative assessment of the impact of socioeconomic status, or mode of delivery, on intervention effectiveness for smoking cessation in pregnancy. We will, however, seek to explore these variables in a qualitative synthesis.

### Strengths

The only known review to currently explore the use of digital interventions for smoking cessation in pregnancy has focused on limited modes of delivery, in particular text messaging and mobile phone apps, and has largely only looked at the content of such interventions [42]. This review aims to go a step further by exploring the effectiveness of all digital interventions and synthesising the research to enhance the body of evidence in this field.

By analysing the BCT content of current digital interventions for smoking cessation in pregnancy, this review aims to address gaps in existing research by assessing the extent to which such interventions are theory-driven and to determine whether theory-driven digital interventions are more likely to be effective for smoking cessation in pregnancy.

### Potential implications

The findings from this systematic review may provide greater understanding amongst those working in digital health and smoking cessation, and smoking cessation in pregnancy, regarding the content of interventions most likely to be effective. Results are likely to be of value for public health and may therefore have the potential to influence the local commissioning of services.

The review findings will also be used in the design of a theory-based intervention aiming to address smoking in pregnancy and will be valuable for others involved in the design of interventions for this population. By guiding the design of new digital interventions which are most likely to be effective for helping pregnant women to quit smoking, this will ultimately provide protective health benefits for both mother and baby.

#### Registration

This systematic review has been registered with PROSPERO, an international prospective register of systematic reviews: http://www.crd.york.ac.uk/PROSPERO/display_record.asp?ID=CRD42016036201. Any changes to the protocol will be amended on this register.

## Additional files

Additional file 1: PRISMA-P checklist. This checklist includes a list of recommended items to include in a systematic review protocol and where these can be found in this protocol. (DOC 82.5 kb)
Additional file 2: CINAHL example full search strategy. This search strategy will be adapted for each database. (DOCX 79.0 kb)

## Abbreviations

ASSIA: Applied Social Sciences Index and Abstracts
BCT: Behaviour change technique
CI: Confidence intervals
CINAHL: Cumulative Index of Nursing and Allied Health Literature
EMBASE: Excerpta Medical Database
MEDLINE: Medical Literature Analysis and Retrieval System
PRISMA: Preferred Reporting Items for Systematic Reviews and Mata-Analyses
PsycINFO: Psychological Information Database
