# Supplementary material for: Are digital interventions for smoking cessation in pregnancy effective? A systematic review protocol
Source: Syst Rev. 2016 Dec 1;5:207. doi: 10.1186/s13643-016-0390-6 (PMC5131429; doi:10.1186/s13643-016-0390-6)
Supplement: Additional file 2: — CINAHL example full search strategy. This search strategy will be adapted for each database. (DOCX 79.0 kb) [file 13643_2016_390_MOESM2_ESM.docx]

**Additional file 2: CINAHL complete search strategy**

| **Subject headings** | **Keyword search** |
| --- | --- |
| 1.Expectant mothers / Pregnancy / Prenatal Care | 1. pregnan* OR “expectant mother*” OR “expectant wom#n” OR prenatal OR antenatal |
| 2. Smoking / Smoking Cessation / Smoking Cessation Programs / Tobacco Products | 2. Smok* OR tobacco OR cigarette* |
| 3. Internet / Social Media/ World Wide Web / Telephone / Smartphone / Mobile Applications / Text Messaging / Electronic Mail / Computers Hand-held/ Computers Portable / Cellular Phone / Telehealth | 3.Telephone OR phone OR “mobile phone” OR “cell* phone” OR smartphone OR app* OR telecommunication OR eHealth OR “e health” OR internet OR web OR “world wide web” OR digital* OR online OR “on line” OR computer OR laptop OR iPad OR tablet OR video OR DVD OR mHealth OR “m health” OR uHealth OR “u health” OR email OR “e-mail” OR “electronic mail” OR “text messag*” OR SMS OR “multimedia messag*” OR MMS OR “social media” |
| 4. Clinical trials / Randomized controlled trials / Quasi-experimental Studies / Experimental Studies | 4. “RCT” OR “randomi#ed control* trial” OR “experimental study” OR “quasi-experimental” OR “clinical trial” OR trial |

*Searching title and abstract*
